# Pathological roles of mitochondrial dysfunction in endothelial cells during the cerebral no-reflow phenomenon: A review

**DOI:** 10.1097/MD.0000000000040951

**Published:** 2024-12-20

**Authors:** Xia Luo, Shaotao Zhang, Longbing Wang, Jinglun Li

**Affiliations:** a Department of Neurology, The Affiliated Hospital of Southwest Medical University, Luzhou, China; b Laboratory of Neurological Diseases and Brain Function, The Affiliated Hospital of Southwest Medical University, Luzhou, China.

**Keywords:** cerebral no-reflow phenomenon, endothelial cells, mitochondrial dysfunctions, ROS

## Abstract

Emergency intravascular interventional therapy is the most effective approach to rapidly restore blood flow and manage occlusion of major blood vessels during the initial phase of acute ischemic stroke. Nevertheless, several patients continue to experience ineffective reperfusion or cerebral no-reflow phenomenon, that is, hypoperfusion of cerebral blood supply after treatment. This is primarily attributed to downstream microcirculation disturbance. As integral components of the cerebral microvascular structure, endothelial cells (ECs) attach importance to regulating microcirculatory blood flow. Unlike neurons and microglia, ECs harbor a relatively low abundance of mitochondria, acting as key sensors of environmental and cellular stress in regulating the viability, structural integrity, and function of ECs rather than generating energy. Mitochondria dysfunction including increased mitochondrial reactive oxygen species levels and disturbed mitochondrial dynamics causes endothelial injury, further causing microcirculation disturbance involved in the cerebral no-reflow phenomenon. Therefore, this review aims to discuss the role of mitochondrial changes in regulating the role of ECs and cerebral microcirculation blood flow during I/R injury. The outcomes of the review will provide promising potential therapeutic targets for future prevention and effective improvement of the cerebral no-reflow phenomenon.

## 1. Introduction

Stroke has attracted significant research attention as an important cause of death and disability; among all stroke cases, ischemic stroke accounts for 70% to 80%.^[1]^ In cases of acute ischemic stroke, blood supply is restored through intravenous thrombolysis or intravascular intervention.^[2]^ Despite the timely and successful endovascular reperfusion treatment, a considerable proportion of patients have had poor functional outcomes. One contributing factor is that blood flow restoration does not mitigate the dysfunction of cellular function and metabolism or structural damage during the reperfusion stage. These issues are instead exacerbated by oxidative stress, and inflammatory cascades, among other factors. This phenomenon is commonly referred to as cerebral ischemia-reperfusion (I/R) injury.^[3]^ The other aspect, known as the “no-reflow phenomenon,” warrants profound scrutiny and greater consideration.

**Figure 1.**
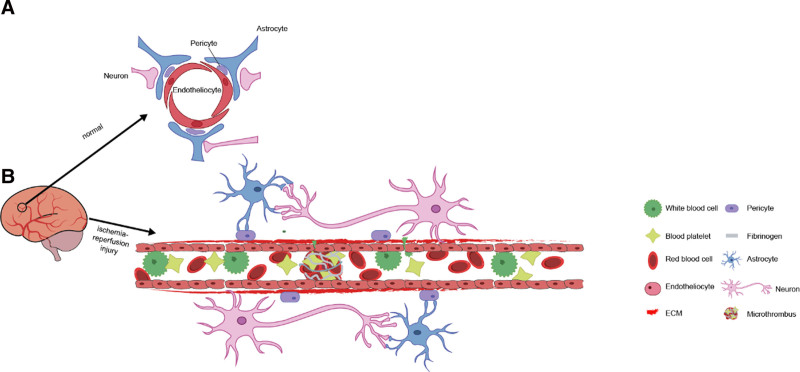
NVU schematic diagram: (A) Under physiological conditions, the NVU structure of cerebral microcirculation includes neurons, neuroglia, pericytes, ECM, and ECs. (B) Under conditions of I/R injury, various components of the NVU change due to ROS production: ECs, pericytes, and neuroglia cells swell; neuronal apoptosis; red blood cells deform; leukocytes adhere to ECs; platelets are activated; fibrinogen is converted into fibrin, forming microthrombi that block small blood vessels. EC = endothelial cells, ECM = extracellular matrix, I/R = ischemia-reperfusion, NVU = vascular neural unit, ROS = reactive oxygen species.

The no-reflow phenomenon is a condition in which a local vessel undergoes spasticity and blockage; blood flow restoration fails in an organ or tissue despite successful restoration to the affected area. Researchers have observed this occurrence in various organs, including the heart,^[4]^ brain,^[5]^ skin,^[6]^ and kidney.^[7]^ At present, the pathophysiological mechanism is broadly recognized to involve microcirculation disturbances.^[8]^ The cerebral microcirculation has a special ultrastructural form, vascular neural unit (NVU), including neurons, neuroglia, pericytes, extracellular matrix, and ECs.^[9]^ The NVU is important for connecting neural activity with blood flow and regulating cerebral blood flow to ensure adequate blood flow to the brain.^[10]^ Figure 1 shows the structure of NVU and pathological changes of its components during the I/R injury.

ECs line the inner surface of the blood vessel, constructing a vast cerebral microvascular network. One of the key roles of vascular ECs is to transport nutrients and oxygen by generating microvasculature.^[8]^ Additionally, ECs serve as the primary barrier against circulating molecules, cells, or pathogens that may enter the bloodstream.^[11]^ But beyond all that, it should be noticed that ECs have peculiar roles, including regulating vascular tone as well as participating in neutrophil recruitment, cell proliferation, angiogenesis, vascular constriction, and relaxation.^[12]^ Hence, ECs play important roles in the maintenance of vascular homeostasis.^[13]^ ECs rely on complex signaling processes responding to environmental changes.^[14]^ They can receive signals from the blood, before translating them into intracellular responses,^[15]^ and further transmit these signals to other layers of the blood vessel wall, hence regulating various physiological activities of other cells.^[16]^

Mitochondria is one of the key platforms to respond to environmental cues in ECs. Although mitochondria are primarily known for their role in energy production through oxidative phosphorylation, emerging research suggests that these organelles also modulate endothelial cell function via various mechanisms.^[17]^ During the I/R injury, mitochondrial dynamics are disturbed, as evidenced by increased fission,^[18]^ decreased fusion,^[19]^ and impaired mitophagy.^[20,21]^ Ultimately, these structural and functional changes translate to changes in endothelial cell behavior, which influence vascular tone, inflammation, and microvascular patency.^[22]^ This review describes the influence of mitochondrial dysfunction on endothelial function and its contribution to microcirculation disturbance in the cerebral no-reflow phenomenon.

## 2. Mitochondria in ECs

Mitochondria, often referred to as “powerhouses of the cell,” are best known for their role in energy production.^[23]^ Compared with other types of cells (mitochondria comprise approximately 28% of liver cells and 32% of cardiomyocytes), ECs contain fewer mitochondria, accounting for only 2% to 6% of the cytoplasmic volume.^[24]^ The function of mitochondria as energy generators may not be prominent in ECs. This is because most glucose is not processed through oxidative phosphorylation in ECs. Instead, more than 80% of adenosine triphosphate (ATP) production occurs through aerobic glycolysis metabolism.^[25]^ The potential benefits of aerobic glycolysis in ECs may include: reducing the production of reactive oxygen species (ROS) generated through oxidative phosphorylation, preserving maximal oxygen levels for delivery to perivascular cells, and facilitating high adaptation to survive, proliferate, and migrate to a hypoxic milieu. It is now widely accepted that mitochondria play a prominent role in responding to environmental signals, and the distribution of mitochondria may influence cellular signal transduction pathways.^[26,27]^ Thus, the limited mitochondria in ECs act as a fundamental platform for regulating the signaling of cellular messengers such as ROS, NO, and calcium ions (Ca^2+^). The precise regulation of these signaling molecules by mitochondria is essential for determining the fate of ECs, including cell proliferation,^[28]^ initiation of apoptosis,^[29,30]^ and modulation of immune inflammation.^[31,32]^

## 3. Mitochondrial ROS in ECs

ROS is 1 major signaling messenger responding to environmental cues in ECs. Under normal physiological conditions, ECs maintain a delicate balance between the production and scavenging of ROS to ensure their signaling functions while at the same time avoiding oxidative damage.^[33]^ ROS, at their levels, act as second messengers of physiological signals by oxidatively modifying various proteins, including receptors, phosphatases, ion channels, and kinases.^[34]^ As a metabolic byproduct, excessive ROS can, however cause DNA damage, lipid peroxidation, mitochondrial damage, and even cell apoptosis.^[35]^ Given the importance of ROS in signal transduction and vascular disease, there is increasing interest in understanding the sources and regulatory mechanisms of mitochondrial ROS in ECs.

### 3.1. Production of mitochondrial ROS in ECs

Oxidative stress is a hallmark of cerebral I/R injury^[36]^ and is linked to ROS accumulation.^[37]^ During cerebral I/R injury, the ECs suffer severe oxidative stress. The electron transport chain (ETC) on the inner mitochondrial membrane (IMM) in vascular ECs generates a colossal amount of ROS when cerebral I/R injury occurs.^[38]^ The major mechanism underlying this phenomenon involves mitochondrial respiratory complexes I and III, with complex I taking prominence in generating most of the ROS precursor superoxide anion (O_2_^−^). These O_2_^−^ thereafter stimulate the production of numerous ROS.^[39]^

Apart from the ETC reducing molecular oxygen to generate O_2_^−^, several other sources of mitochondrial ROS have been identified. Significant ROS generation in ECs occurs due to oxidase system activation and antioxidant enzyme system inhibition.^[33]^ Nicotinamide adenine dinucleotide phosphate oxidase (NOX) is a major enzyme responsible for ROS generation in ECs.^[40]^ It comprises 4 subtypes: NOX1, NOX2, and NOX5,^[41]^ which produce O_2_^−^; NOX4 produces hydrogen peroxide and is located on the IMM and demonstrates high expression in ECs. It activates various downstream signaling pathways related to ROS, including p53,^[42]^ nuclear factor-kappa B (NF-κB),^[43]^ Src kinase,^[44]^ and hypoxia-inducing factor 1.^[45]^ Under I/R conditions, xanthine oxidase is activated and implicated in purine metabolism, which is responsible for generating O_2_^−^ and hydrogen peroxide to produce ROS.^[46]^ These ROS promote the attachment of neutrophils in the bloodstream to ECs, leading to increased ROS production.^[47]^ This process is more prominent in ECs because xanthine oxidase is abundant in ECs.^[48]^ In response to the stimuli of I/R injury, another source of ROS in ECs is catecholamine catabolism regulated by the monoamine oxidase, located in the outer mitochondrial membrane (OMM).^[47]^ Moreover, the growth factor adaptor protein p66Shc participates in ROS generation in ECs. Proapoptotic signals migrate p66Shc into the mitochondrial intermembrane space and then p66Shc generates hydrogen peroxide by oxidizing cytochrome C (CytC).^[49,50]^ Lastly, reduced levels of antioxidants in the mitochondrial matrix such as superoxide dismutase 2, glutathione peroxidase, and catalase also contribute to ROS accumulation.^[40,44]^

### 3.2. Regulation of mitochondrial ROS production in ECs

Many mechanisms modulate mitochondrial ROS production in ECs. Among these, nitric oxide (NO) and intracellular Ca^2+^ have been extensively noticed and studied. NO is a diffusible gas synthesized by a variety of NO synthases regulating ROS production and vasomotor function.^[30]^ In ECs, endothelial nitric oxide synthase (eNOS) catalyzes the production of L-citrulline and NO from L-arginine and O_2_^−^.^[51]^ When I/R injury occurs, ROS is accumulated in ECs and reduces the content of NO in ECs. On the 1 hand, polymerase (poly ADP-ribose) activation reduces the activity of glyceraldehyde 3-phosphate dehydrogenase, further activating protein kinase C and subsequently NF-κB.^[51,52]^ This causes a reduction in NO production due to downregulated expression of eNOS. On the other hand, ROS derived from NoX oxidizes NO to form peroxynitrite, further reducing NO availability.^[53]^ NO has a bidirectional regulatory role under physiological conditions, depending on the oxygen concentrations and the cellular redox state. NO can reversibly bind to and temporarily block complex IV in lower amounts,^[54]^ thereby controlling mitochondrial respiration and facilitating the release of mitochondrial ROS. Besides, NO can directly and chemically react with other molecules to form peroxynitrite (ONOO), stabilize CytC, clear O_2_^−^, and reduce complex I activity,^[55,56]^ causing a decrease in mitochondrial ROS production. Thus, in I/R injury conditions, the reduced endothelial NO bioavailability may promote excessive production of ROS by eliminating the NO effect on inhibition of mitochondrial complex I.

Ca^2+^ acts as a secondary messenger to modulate energy metabolism, and mitochondrial membrane potential, and influence ROS generation.^[57]^ This process ensures the proper functioning of ECs. Mitochondria in ECs participate in calcium homeostasis.^[58]^ Through mitochondrial calcium uniporter and associated regulatory proteins, mitochondria promote the buffering of intracellular calcium levels by sequestering excessive Ca^2+^ into their matrix.^[59]^ During I/R injury, Ca^2+^ overload caused by mitochondrial dysfunction is a key pathological event in ECs.^[60]^ However, the controversy surrounding the effects of elevated Ca^2+^ on mitochondrial ROS production is a topic of significant interest and debate in the field of cellular biology and physiology. In general, in the condition of mitochondrial membrane depolarization, or there is some degree of mitochondrial electron transport inhibition, Ca^2+^ appears to have increased mitochondrial ROS production.^[61]^ Experimental findings have suggested several mechanisms: the increased Ca^2+^ contributed to the eNOS activation via the Ca^2+^/CaMKII/AMPK/p38MAPK type/Akt signal cascade,^[62]^ and promoted the production of NO to inhibit to block mitochondrial respiratory complex IV.^[54]^ Ca^2+^ controls membrane potential, regulates mitochondrial ATP production by modulating the activity of enzymes involved in the tricarboxylic acid cycle and oxidative phosphorylation, and enhances electron flow into the mitochondrial oxidative respiratory chain.^[63]^ Ca^2+^ changes MICU1 and the cellular mitochondrial cristae junction structure, releasing CytC from the IMM.^[64]^ The recognized function of CytC mainly acts as the electron carrier and helps the electronic in the mitochondrial respiratory chain complex III transfer to complex IV.^[61]^ When the CytC is released from the respiratory chain, the content of O_2_^−^ and H_2_O_2_ will increase sharply, promoting the generation of ROS in ECs.^[40]^ Interestingly, researchers have found that moderate increases in mitochondrial Ca^2+^ can enhance mitochondrial metabolism and ATP production, potentially reducing ROS levels by improving the efficiency of the ETC.^[65]^

### 3.3. Contribution of mitochondrial ROS in ECs

Emerging evidence suggests that ROS in vascular ECs plays a nuanced and essential role in modulating vascular function and signaling. High levels of ROS participate in many interrelated mechanisms that promote endothelial apoptosis and microcirculation disorders.

#### 3.3.1. Regulate the endothelial apoptosis

Mitochondria in ECs act as central mediators of apoptosis and regulate cell survival.^[66]^ Under normal conditions, mitochondria release factors promoting cell survival and prevent apoptosis.^[67]^ Nevertheless, in acute I/R injury, ROS surges and accumulates in ECs due to oxidative stress. ECs exposed to high levels of ROS undergo a series of molecular events that lead to apoptosis, or programmed cell death.^[68]^ ROS induces endothelial apoptosis by activating 3 major apoptotic signaling pathways: the mitochondrial pathway, the death receptor pathway, and the endoplasmic reticulum (ER) pathway.^[69]^

Caspases play a special role in the intrinsic mitochondrial apoptotic and death receptor apoptotic pathways, and their activation is one of the hallmarks of apoptosis. Under the oxidative stress condition, ROS accumulation in ECs leads to mitochondrial membrane damage and the release of CytC from IMM into the cytoplasm. Subsequently, CytC interacts with apoptosis protease activating factor 1 and caspase-9, which in turn activates caspase-3 and caspase-7, culminating in the fragmentation of DNA and cellular demise.^[70]^ The activation of caspase-3 leads to the cleavage of bid (Bcl-2 family protein), further mediating the release and transport of CytC.^[71]^ In addition, ROS can activate BH3-only proteins to inhibit the antiapoptotic factor BCL-2 and activate the proapoptotic factors such as Bax and Bak, inducing the opening of mitochondrial permeability transition pore (mPTP) to trigger apoptosis.^[72–74]^

The mitogen-activated protein kinase (MAPK) family includes extracellular signal-regulated kinases, c-Jun N-terminal kinases (JNKs), and p38 MAPKs, and plays a key role in mediating cellular responses to environmental stresses and intracellular conditions.^[75]^ Among these, the JNK signaling pathway is particularly sensitive to ROS and plays a pivotal role in apoptosis, or programmed cell death.^[76]^ The JNK activated by ROS can lead to both intrinsic and extrinsic apoptotic signaling. JNK can directly activate caspase-9 and phosphorylate Bcl-2 family members,^[77]^ promoting their proapoptotic functions and further enhancing mitochondrial outer membrane permeabilization. On the other hand, JNK can modulate the apoptotic pathway by influencing the expression and function of death receptors and their ligands, such as Fas (CD95) and tumor necrosis factor receptor 1. Upon ligand binding, these receptors recruit adaptor proteins like Fas-associated death domain protein and procaspase-8, forming the death-inducing signaling complex.^[75]^

In recent years, research has established a significant link between ROS-induced apoptosis and ER stress. The interplay between ROS-induced apoptosis and ER stress in ECs is complex and multifaceted. Both processes can amplify each other, leading to a vicious cycle of oxidative damage and cellular dysfunction. ROS can oxidize lipids and proteins in the ER, leading to the formation of misfolded proteins that accumulate within the organelle, thereby triggering a series of signaling pathways known as the unfolded protein response (UPR).^[78,79]^ The UPR involves the activation of 3 main transmembrane proteins: protein kinase RNA-like endoplasmic reticulum kinase, inositol-requiring enzyme 1, and activating transcription factor 6.^[80]^ The high levels of ROS induce prolonged ER stress, the UPR can shift towards a proapoptotic state by inhibiting antiapoptotic proteins and inducing the expression of proapoptotic proteins. Additionally, inositol-requiring enzyme 1 can activate the JNK pathway, which also promotes apoptosis.^[81]^

#### 3.3.2. Regulate the microcirculation

Microcirculation refers to the smallest blood vessels in the circulatory system, including capillaries, arterioles, and venules. Excessive ROS causes endothelial injury and can regulate microcirculation through multiple mechanisms during cerebral I/R injury, including vascular tone, micro-thrombosis, angiogenesis, and inflammatory response.

High levels of ROS in ECs play a critical role in regulating vascular tone and ultimately cause microcirculation vasospasm, restricting blood flow to brain tissue during reperfusion. One of the key mechanisms is by affecting the production and activity of NO. NO is a potent vasodilator produced by ECs and it plays a crucial role in maintaining vascular homeostasis. As mentioned in Section 3.2, ROS generation results in a decrease in NO, contributing to vasoconstriction.^[82]^ Similarly, ROS can inhibit the activity of endothelial-derived hyperpolarizing factor^[83]^ and prostacyclin,^[84]^ both of which contribute to vasodilation. In addition, ROS can influence vascular contraction and relaxation by modulating the activity of other signaling molecules and ion channels. ROS can activate protein kinase C^[85]^ and MAPKs,^[86]^ leading to increased phosphorylation of myosin light chain and subsequent contraction of vascular smooth muscle cells by increasing intracellular calcium levels. ROS can also directly interact with and modify the function of ion channels, such as potassium channels,^[87]^ which play a role in regulating the membrane potential and contractility of smooth muscle cells. Furthermore, ROS can induce the expression of genes that promote vascular contraction and inhibit those that promote relaxation. This can occur through the activation of transcription factors such as nuclear NF-κB and activator protein-1,^[88]^ which are sensitive to oxidative stress. By promoting the expression of contractile proteins and inhibiting the expression of relaxant proteins, ROS can contribute to the maintenance of high vascular tone.

Excessive ROS induce the accumulation of blood components and block microcirculation. ECs typically lack adhesion molecules and thrombosis factors, preventing neutrophil and platelet accumulation in the bloodstream. However, leukocyte adhesion molecules are upregulated when ECs undergo pathological changes due to the massive production of ROS, thus stimulating neutrophils and macrophages to accumulate and adhere to the inner wall of blood vessels.^[89]^ Neutrophil adhesion induces subsequent ROS release, thereby creating a feedback loop that reinforces this process^[90]^ as well as intensifying cellular inflammation and blood cell aggregation, ultimately resulting in the blockage of microcirculatory vessels. Furthermore, the platelet aggregation pathway associated with the GPIIb/IIIa receptor is activated due to reduced NO synthesis and increased intracellular calcium levels, resulting in microthrombi formation.^[91]^

Oxidative stress caused by ROS has a multifaceted impact on ECs, leading to delayed growth, cellular senescence, and apoptosis.^[92]^ In ECs, the mechanism by which ROS induces senescence in ECs involves the activation of several pathways, including the p53 and p16INK4a pathways,^[93]^ which are key regulators of cell cycle progression and senescence. Furthermore, the evaluated ROS can inhibit the activation of vascular endothelial growth factor receptors,^[94]^ which are critical for the initiation of angiogenic signaling. Additionally, ROS can interfere with the signaling pathways that regulate cell migration, such as the Rho family of small GTPases,^[95]^ which are key regulators of the cytoskeleton. The cumulative effect of these changes is a reduction in the vascular bed, which compromises the ability of the microvasculature to mediate compensatory regulation of blood flow. This can lead to tissue ischemia and hypoxia, further exacerbating the production of ROS and creating a vicious cycle of oxidative stress and vascular damage.

Notably, ROS plays a pivotal role in the pathophysiology of vascular diseases by triggering a series of inflammatory responses.^[96]^ In ECs, ROS acts as a critical signaling molecule that initiates and propagates inflammatory responses. They interact with various intracellular sensors and signaling pathways, such as the NF-κB and MAPK pathways,^[97]^ which are central to the regulation of inflammatory gene expression. Through these pathways, ROS stimulate the production and secretion of numerous inflammatory mediators, including cytokines (such as tumor necrosis factor-alpha and interleukin-6), chemokines, and adhesion molecules.^[98]^ The inflammatory response induced by ROS in ECs has several downstream effects on vascular function. Inflammatory cytokines and ROS can activate endothelial receptors and signaling molecules that lead to the constriction of blood vessels, a process that is crucial for regulating blood flow and pressure.^[99]^ Another significant consequence of inflammation induced by ROS in ECs is the alteration in endothelial permeability. Inflammatory mediators can disrupt the integrity of the endothelial barrier, leading to increased permeability and the leakage of plasma components into the surrounding tissues.^[100]^ This increased permeability can cause vascular swelling and compression of the vascular lumen, further impairing blood flow and contributing to microcirculation disorders.

## 4. Mitochondrial dysfunction in ECs

**Figure 2.**
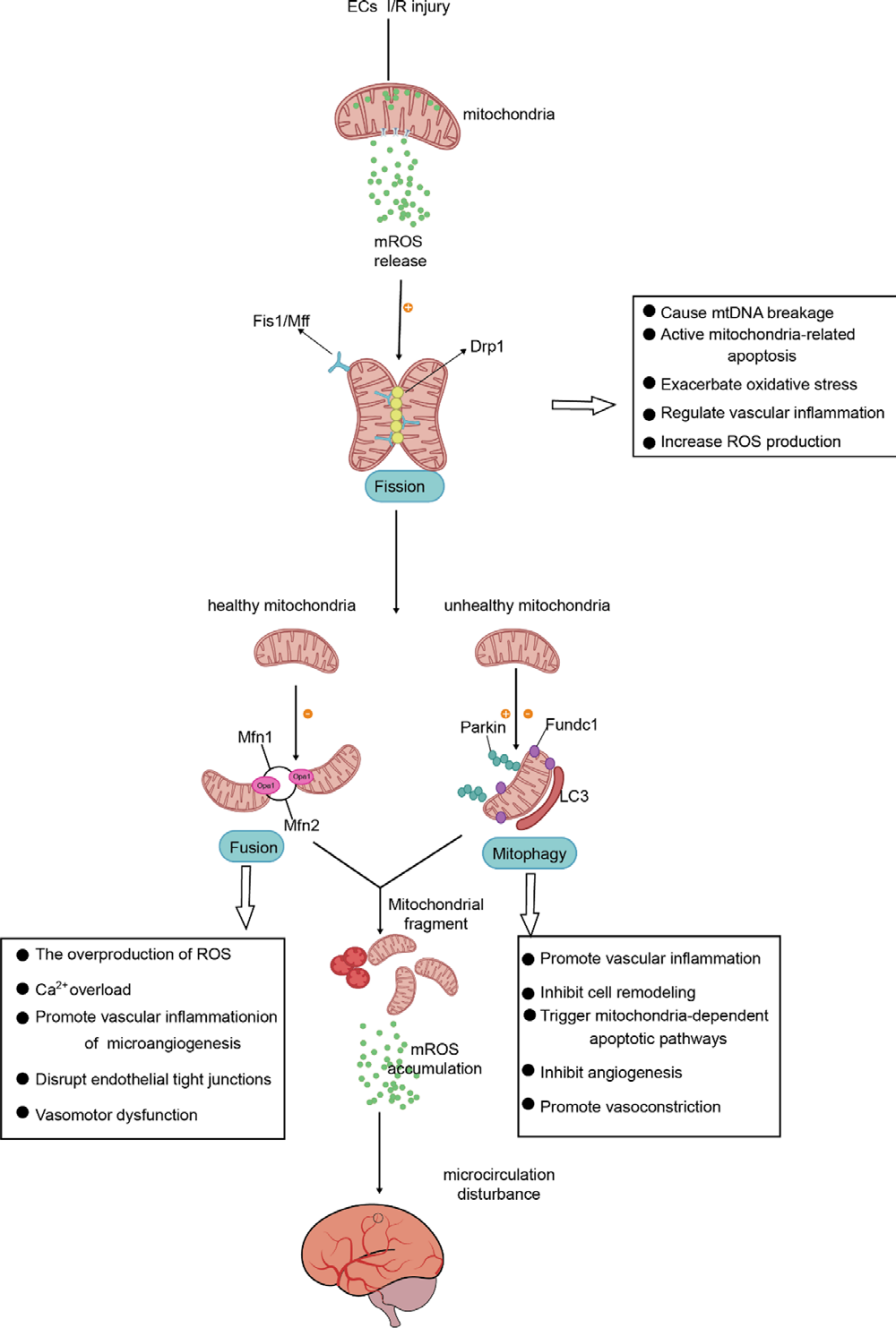
Mitochondrial dysfunctions in ECs during cerebral I/R injury: there is an increase in mitochondrial fission, a decrease in mitochondrial fusion, and a dual level of mitophagy inhibition. They influence endothelial cell fate through various mechanisms to regulate cerebral microcirculatory blood flow. EC = endothelial cells, I/R = ischemia-reperfusion, ROS = reactive oxygen species.

Mitochondria undergo continuous fission, fusion, and autophagy processes to reshape mitochondrial morphology in response to physiological and pathological cues.^[101,102]^ Under I/R injury conditions, regulatory mechanisms controlling the mitochondrial dynamic network mentioned above are imbalanced. Figure 2 describes the mitochondrial dysfunction in ECs and mechanisms regulating endothelial function to cause regional microcirculatory obstruction.

### 4.1. Upregulated mitochondrial fission

Under I/R injury conditions, excessive ROS increases mitochondrial fission. Several fragmented and dysfunctional mitochondria finally accumulate in the cytoplasm, a major contributor to endothelial cell injury.^[103]^ During mitochondrial fission, the actin-myosin cytoskeleton initiates an initial contraction event.^[103]^ Furthermore, dynein-associated protein-1 (Drp1) participates in remodeling the mitochondrial membrane.^[103]^ Drp1, upon activation, relocates to the OMM and assembles into oligo-rings. guanosine triphosphate hydrolysis causes ring contraction, resulting in the separation of mitochondria into 2 distinct organelles.^[104]^ There have been extensive studies on Drp1 phosphorylation at specific sites, including Ser616,^[105]^ Ser637,^[106]^ Ser656,^[107]^ Ser600,^[108]^ and Ser693.^[109]^ Drp1 phosphorylation at Ser616 promotes the in vitro assembly of Drp1 particles into membrane oligomers and the formation of potential splitting rings.^[110]^ In a study of hypoxia-induced hippocampal neuron damage in rats, Drp1 was phosphorylated primarily at Ser616 and dephosphorylated at Ser637, fragmenting mitochondria and disrupting mitochondria balance in the cells.^[105]^ Therefore, the ratio of phosphorylation at Ser616/Ser637 acts as an indicator for Drp1 activation and mitochondrial fission. Significant research has been performed to assess the impact of I/R injury on the process of mitochondrial fission in neurons; however, research on microvascular ECs remains limited.

The mitochondrial transmembrane potential decreases, and CytC leaks in mitochondria with increasing mitochondrial fission.^[111]^ However, whether fission is a necessary factor in mitochondria-dependent apoptosis remains debatable. Drp1 levels vary in ECs at various stages of IR injury. Under hypoxic conditions, Drp1 phosphorylation is barely detectable. Notably, the level of Drp1 phosphorylation tends to increase upon reoxygenation, causing an increase in pathological mitochondrial fission within the cell.^[112]^ Due to limited mitochondria, ECs rely on anaerobic glycolysis for energy production. Therefore, the hypoxic environment has minimal impact on mitochondrial function and energy production in ECs. Mitochondria, instead act as sensitive receptors to activate various antioxidant systems and remove ROS during the early stages of hypoxia,^[113]^ which may justify why mitochondrial fission is not induced by oxidative stress. Nonetheless, the oxidative stress damage is intensified with ROS release during the reperfusion stage. ROS accumulation disrupts the electron transfer process of the mitochondrial oxidative respiratory chain. Subsequently, H^+^ transmembrane gradient formation is influenced, causing decreased mitochondrial membrane potential. To remove the dysfunctional and damaged mitochondria in ECs, the Drp1 pathway is activated to improve mitochondrial fission and exacerbate cellular oxidative stress damage.^[114]^ During reperfusion, the restoration of blood perfusion and fluid shear forces exerted on ECs also promote mitochondrial fission.^[115]^

Recent research findings have shown that excessive mitochondrial fission can have detrimental effects on ECs by causing mitochondrial DNA (mtDNA) breakage, activating mitochondria-related apoptosis, and exacerbating oxidative stress. Excessive fission can first cause double-strand breaks in the mtDNA of ECs, damaging mtDNA replication and transcription. mtDNA damage directly influences the role of the mitochondrial respiratory complex. This damage increases ROS production, further compromising mitochondrial function, hence creating a vicious cycle of damage and dysfunction.^[116]^ Second, in oxidative stress-induced excessive fission, drp1 detects mPTP channels by binding to BAX-PiC, followed by LRRK2 and HK2 inactivation. As a result, the mPTP pore structure is destroyed and opened.^[117]^ With ROS accumulation, cardiolipin peroxidation loses its affinity for CytC. CytC then detaches from the IMM and moves to the cytoplasm, activating caspase-3 and caspase-9-related apoptotic pathways, ultimately resulting in EC apoptosis. Furthermore, mitochondrial fission also promotes the regulation of endothelial inflammation,^[118]^ hence contributing to endothelial dysfunction during reperfusion injury. Nonetheless, more direct evidence from animal models of cerebral I/R injury is necessary to fully understand this phenomenon.

### 4.2. Downregulated mitochondrial fusion

Unlike mitochondrial fission, mitochondrial fusion promotes efficient transport and distribution of mitochondrial contents, enabling information exchange in the mitochondrial network via sequential merging of the OMM and IMM.^[119]^ The fusion process is a protective mechanism in mitochondrial dynamics that helps maintain mitochondrial homeostasis under different stresses. Under pathological conditions including excessive oxidative stress and energy metabolism imbalance, the balance between mitochondrial fission and fusion is often tilted to 1 side.^[19]^

Since mitochondria have double membranes, their fusion involves a coordinated merger of OMM and IMM. This process is promoted by 3 major proteins, including mitofusin 1 (Mfn1), mitofusin 2 (Mfn2), and optic nerve atrophy 1 (Opa1). Of note, Mfn1/2 are specific GTPases in the OMM, whereas Opa1 is situated in the IMM. Despite not having a full understanding of the intricate molecular process underlying mitochondrial fusion, recent studies have shown that guanosine triphosphate binding and hydrolysis cause structural changes in the transmembrane region of Mfn. Mfn1/2 forms oligomeric complexes between 2 adjacent mitochondria.^[120]^ Subsequently, phospholipid membrane D mediates the hydrolysis of cardiolipin to phosphatidic acid, promoting outer membrane bending; Mfn further binds mitochondria to achieve mitochondrial outer membrane fusion.^[121]^ The fusion mechanism of IMM is more complex than that of OMM and primarily regulated by Opa1.^[122]^ Respiratory complex dysfunction in the IMM caused by oxidative stress causes a ROS increase and a decrease in manganese superoxide dismutase. Additionally, mitochondrial fusion is downregulated.^[123]^ Multiple laboratory studies have confirmed that mitochondria blocking the fusion ultimately causes reduced proliferation, increased apoptosis, and dysfunction of ECs.^[124]^ The present study speculates that the reduction of mitochondrial fusion is associated with endothelial dysfunction during cerebral I/R injury.

During I/R injury, possible mechanisms under which mitochondrial fusion regulates endothelial dysfunction are as follows: First, there is an imbalance between mitochondrial fission and fusion, causing impaired mitochondrial performance and ROS overproduction.^[88]^ Second, considerable fluid shear stress mechanically damages ECs during the initial stage of I/R injury, thereby promoting cell death. Moreover, intracellular calcium levels affect mitochondrial fusion activity, further impacting endothelial function.^[115]^ Third, a reduction in mitochondrial fusion promotes vascular inflammation by activating the NLR family containing pyrin domain containing 3 (NLRP3) inflammasomes.^[100,125]^ When fusion is damaged, the resulting endothelial edema and vascular can oppress diastolic dysfunction of microcirculation, further limiting blood flow to the reperfusion phase, and forming a vicious cycle.^[126]^ Fourth, the Mfn2 upregulation promotes mitochondrial fusion, addressing the disruption of connectivity between ECs,^[127]^ which improves activation against blood clotting, reducing microthrombus formation. Lastly, inhibition of fusion inhibits eNOS phosphorylation, decreases NO production,^[128]^ and impairs ECs-dependent vasodilation as well as vasoconstriction. It is important to acknowledge that the aforementioned evidence is primarily based on in vitro experiments. Therefore, additional investigations are necessary to fully understand the role of mitochondrial fusion in cerebral I/R injury and identify promising targets for intervention.

### 4.3. Dysregulated mitophagy

As an intracellular self-degradation system, mitophagy is stimulated to eliminate dysfunctional mitochondria.^[129]^ Studies indicate that excessive mitophagy induced by certain adaptor proteins can cause mitochondrial depletion and compromise cellular function, whereas basal or moderate levels of autophagy are crucial for cellular health. Mitochondrial autophagy below the baseline physiological level can be harmful when ECs are subjected to oxidative stress. Inadequate autophagy fails to clear dysfunctional mitochondria, ultimately causing a buildup of ROS and release of apoptotic factors, as a consequence of endothelial dysfunction and apoptosis. Thus, maintaining a suitable level of mitophagy is key for the survival and function of ECs. The dual effect of mitophagy on neurons in cerebral IR injury has received intense debate. Excessive mitophagy can cause the removal of functional mitochondria, resulting in energy depletion, impaired cellular respiration, and increased susceptibility to apoptosis. However, studies suggest that an adaptive increase in mitophagy can be beneficial during hypoxia or nutrient deprivation, where mitochondria recycling promotes cellular energy preservation and survival.

Communication between mitochondria and lysosomes involves several splice proteins, including Bnip3, Fundc1, Nix, and Parkin. The LC3 protein family is an important player in the mitophagy process; the LC3 protein family exists in 2 forms, that is, LC3-I and LC3-II; LC3-I is the cytosolic form, whereas LC3-II is associated with autophagosomal membranes. LC3-I conversion to LC3-II is a key step in mitophagy initiation.^[93]^ Recent research results have reported that the Pink1-Parkin pathway is broadly considered the most important pathway. Parkin-induced mitophagy operates without specific receptors. Noteworthy, Bnip3 can preserve PINK1 stability by preventing its proteolytic cleavage. Additionally, both Nix and Bnip3 participate in regulating Parkin recruitment.^[130,131]^ This suggests interconnectedness between mitotophagy receptors, rather than complete independence.

Mitophagy expression varies at different stages of I/R injury. During the ischemic stage, mitophagy is upregulated to eliminate damaged mitochondria.^[20]^ Nonetheless, mitophagy is suppressed when the reperfusion stage occurs, causing a buildup of impaired mitochondria as well as increased endothelial cell apoptosis and necrosis.^[21]^ Different adaptor proteins play distinct roles in regulating mitophagy, demonstrating complexity in their roles and effects on endothelial cell fate. One such adaptor protein is Fundc1, which helps in preserving mitochondrial integrity and reducing ROS production.^[132]^ Other adaptor proteins, including Pink1-Parkin become over-activated during reperfusion in ECs^[133]^; however, their roles appear more dualistic. The majority of existing research focuses on unraveling the regulatory mechanisms of individual adaptor proteins. However, it is important to decipher the complex regulatory mechanisms underlying mitophagy induction by different adaptor proteins, as well as understand the dual role of mitophagy in various stages of ECs and microcirculation disorders; this is vital for developing targeted interventions to modulate the progression of cerebral microvascular IR injury.

Mitophagy is an important process for ECs in preserving their function and survival under various stress conditions. One vital aspect is its role in regulating ROS levels. Studies have shown that mitophagy induced by hyperglycemia or hyperlipidemia conditions can delay endothelial cell aging by reducing ROS production.^[134]^ Furthermore, inhibiting mitochondrial hyperautophagy via the Pink1-Parkin pathway can effectively prevent ROS overproduction by the respiratory complex in mitochondria.^[134]^ This helps preserve mitochondrial function, reducing cellular oxidative stress, and protecting cardiac microvascular ECs, thereby alleviating coronary reflow. Mitophagy helps in remodeling ECs during times of oxidative stress. This remodeling is catalyzed by a series of events involving the SIRT3-FOXO3 pathway and inhibition of mitochondrial lipid peroxidation.^[135]^ These processes are fundamental for preserving the integrity of the microvasculature, which is critical for overall vascular health. Moreover, excessive mitophagy induced by Parkin results in the opening of mPTP and CytC release, ultimately inducing mitochondria-dependent apoptosis of ECs.^[136]^ This mechanism highlights the importance of properly regulating mitophagy to prevent adverse cellular outcomes in ECs. Furthermore, Zhou et al^[112]^ discovered that mitophagy participates in microangiogenesis. Additionally, they noted increased levels of inflammatory factors, which promote excessive microvascular permeability. ECs from infarcted heart tissue showed significantly higher levels of adhesion factors important for thrombosis in microcirculation. Mitophagy suppression exacerbated the effects mentioned above, underscoring the involvement of mitophagy in preserving the integrity of the endothelial barrier and microvessel function. In pathological conditions where mitophagy is compromised, the balance between eNOS and ET-1 can be disrupted.^[137]^ This disruption can lead to an overproduction of ET-1 relative to NO. Of note, ET-1 is a potent vasoconstrictor that directly acts on smooth muscle cells, increasing intracellular calcium levels and promoting vasoconstriction.^[138]^ Moreover, the interplay between mitophagy and ET-1 extends beyond the vascular system. ET-1 has been implicated in various cellular processes, including inflammation, fibrosis, and cell proliferation.^[139]^ Mitophagy can affect the metabolic state of the cell and the availability of substrates necessary for ET-1 synthesis.^[140]^ Additionally, mitophagy can impact the signaling pathways that regulate ET-1 expression, such as the MAPK^[141]^ and NF-κB pathways.^[142]^

## 5. Conclusion

In conclusion, this review describes the endothelial dysfunctions induced by mitochondrial dynamics and how they influence microvascular blood flow regulation in cerebral I/R injury. As noted, physiological ROS signaling induces functional changes in ECs for adequate blood flow by regulating vascular tone and promoting angiogenesis. Under oxidative stress, mitochondrial dynamic changes disrupt homeostasis, further intensifying ROS accumulation, which promotes the onset and progression of vascular disorders. Moreover, microcirculation faces challenges in obtaining adequate blood supply due to factors including inflammation-induced blood vessel stimulation, impaired vasomotor regulation, and disturbed vascular remodeling. These factors exacerbate hypoxia in surrounding cells. Despite the abundant experimental evidence emphasizing the important role of mitochondria in preserving endothelial function, there is a significant absence of translation in clinical practice. Therefore, future studies on microvascular endothelial mitochondrial dysfunction are required to provide important insights into the pathogenesis of cerebral no-reflow phenomenon and promote the development of novel treatment approaches to mitigate microcirculation disease and associated complications.

## Author contributions

**Conceptualization:** Xia Luo, Jinglun Li.

**Methodology:** Xia Luo, Jinglun Li.

**Formal analysis**: Xia Luo.

**Investigation:** Xia Luo, Shaotao Zhang, Longbing Wang.

**Data curation**: Xia Luo, Shaotao Zhang, Longbing Wang.

**Supervision:** Jinglun Li.

**Project administration**: Xia Luo, Jinglun Li.

**Writing – original draft**: Xia Luo, Jinglun Li.

**Writing – review & editing**: Xia Luo, Shaotao Zhang, Jinglun Li.

**Visualization:** Xia Luo, Shaotao Zhang.

**Funding acquisition:** Jinglun Li.
